# Clinicopathological Characteristics of Four Cases of Adrenal Myelolipomas: A Rare Surgical Entity

**DOI:** 10.4021/wjon600w

**Published:** 2013-01-04

**Authors:** Dionysios Dellaportas, Athanasios Tsagkas, Despoina Myoteri, John Contis, Agathi Kondi-Pafiti

**Affiliations:** a The 2nd Department of Surgery, Aretaieion University Hospital, Athens, Greece; b Department of Pathology, Aretaieion University Hospital, Athens, Greece; c Department of Pathology, ‘Tzaneion’ General Hospital, Piraeus, Greece

**Keywords:** Adrenal gland, Myelolipoma, Adrenalectomy

## Abstract

Myelolipomas are unusual benign tumors or tumor-like lesions, composed of hematopoietic cells and mature adipose tissue. They usually are asymptomatic and behave as non-functioning, unilateral, small adrenal tumors often found incidentally on imaging studies. We report the clinicopathological characteristics of four cases of adrenal myelolipomas treated in our hospital, worth to mention because of their rarity and their significant size. Myelolipomas were first described by Gierke in 1905, and the term *myelolipoma* was coined by Oberling in 1929. The adrenal gland is the most common site, but myelolipomas are also rarely present in extra-adrenal sites, including the pelvis, mediastinum, retroperitoneum, and paravertebral region, as an isolated soft tissue mass. These tumors account for 2.6% of the primary adrenal masses with equal sex distribution and in our reviewed material of a decade they constitute about 5% in a series of surgically treated adrenals for various neoplastic processes of the adrenals. Although usually small in size, in our series a remarkable large size of the tumors examined was observed, ranging from 7 - 15 cm. Adrenal myelolipoma is often an “incidentaloma”, since its diagnosis is frequently based on autoptic findings or made during surgical interventions and imaging procedures performed for other purposes, as happened in our cases.

## Introduction

Myelolipomas are unusual benign tumors or tumor-like lesions, composed of hematopoietic cells and mature adipose tissue. Most cases develop in the adrenal glands but extra-adrenal lesions are also reported, arising in the retro-peritoneum, mediastinum, liver and lungs, as well as in heterotopic adrenals [1]. They usually behave as non-functioning, unilateral, small adrenal tumors and are often found incidentally on imaging studies, without any symptomatology [2].The average age of diagnosis is about 50 years, and there is equal sex distribution. The incidence at autopsy material is 0.08-0.2% [1]. In this study we report the clinicopathological characteristics of four cases of adrenal myelolipomas treated in Aretaieion University Hospital, worth to mention because of their rarity and their significant size, which exceeded at least two times the mean currently reported size of myelolipomas. A brief literature review is also attempted as well.

## Case Report

### Case 1

A 57-year-old male complaining of chronic back pain underwent an abdominal computed tomography (CT-scan), which revealed a large retroperitoneal tumor probably of adrenal origin. Open surgical approach was considered appropriate as long as malignancy could not be excluded definitely. Right adrenalectomy was finally performed and pathological examination showed a 7 × 5 × 4 cm adrenal myelolipoma composed of mature adipose tissue and hematopoietic cells and weighting 250 g. On the tumor periphery a narrow zone of healthy adrenal tissue was observed. The patient was discharged on the fifth postoperative day, and remains disease free three years later.

### Case 2

A 40-year-old white woman with severe anemia of unknown origin was investigated because of enlarged spleen. During investigation an abdominal CT-scan showed a large right adrenal tumor ranging about 15 cm, which was an incidental finding. After proper hormonal laboratory studies, and exclusion of pheochromocytoma and Cushing’s syndrome, operative treatment was decided. The patient underwent an uneventful open right adrenalectomy. Pathology report revealed a 15 × 7 × 6 cm yellow tan, round and firm mass, weighting 420 g, which microscopically consisted of mature adipose tissue mixed with elements of bone marrow, finally diagnosed as myelolipoma. Patient is healthy and disease free 5 years after surgery.

### Case 3

A 45-year-old woman with a personal history of cholelithiasis, arterial hypertension and elevated urine cathecholamines was admitted to our hospital for further imaging diagnostic examinations and treatment. An abdominal CT-scan revealed an about 10 cm right adrenal mass, which was considered to be a pheochromocytoma. After appropriate preoperative preparation with the use of phenoxybenzamine and propranolol the patient was operated in an open fashion. Pathological examination showed a large adrenal tumor weighting 185 g tumor and measuring 10 × 8 × 3.5 cm. Grossly the tumor was yellow-brown, with elastic consistency and microscopically presented the typical myelolipoma features.

### Case 4

A 74-year-old woman with the history of breast carcinoma, under treatment, was diagnosed with a large adrenal tumor, about 8 cm in diameter during routine follow-up ultrasound of the abdomen. Abdominal CT-scanning followed and the adrenal tumor was radiologically heterogeneous with non-specific imaging features. Surgical intervention was decided because of inability to safely exclude malignancy, either primary or metastatic. Pathological examination of the specimen showed a myelolipoma weighting 120 g and atrophic changes of the adrenal cortex.

## Discussion

Myelolipomas are rare benign tumors composed of mature adipose tissue and hematopoietic elements. They were first described by Gierke in 1905, and the term *myelolipoma* was coined by Oberling in 1929 [2]. The adrenal gland is the most common site, but myelolipomas are also rarely present in extra-adrenal sites, including the pelvis, mediastinum, retroperitoneum, and paravertebral region, as an isolated soft tissue mass [3].

The exact etiology of adrenal myelolipomas is still unknown and their histogenesis is uncertain. According to some authors, the tumor develops from residual embryonic cells of the bone marrow that, after embolization reach the adrenal gland. According to a recent theory, myelolipoma originates from a metaplastic process of the reticuloendothelial cells within adrenal capillaries. Some authors even consider the development of adrenal myelolipomas as a response to various endocrine stimulations [4]. This hypothesis is confirmed by the autopsy findings of myelolipomas in patients who died as a result of chronic systemic diseases. Other contemporary authors have speculated about a stressful lifestyle and an unbalanced diet as factors that may be involved in the pathogenesis of this tumor [5] and recent data support the theory that myelolipomas are true neoplasms.

These tumors account for 2.6% of the primary adrenal masses with equal sex distribution [6] and in our reviewed material of a decade they constitute about 5% in a series of surgically treated adrenals for various neoplastic processes of the adrenals. Although most frequently encountered in individuals between fifth and seventh decades, they are also reported in children [7]. They are usually small (< 5 cm), but giant myelolipomas reaching up to 34 cm are also known to occur [8]. In our series, it is remarkable the large size of the tumors examined, ranging from 7 - 15 cm. Unilateral myelolipomas are more common; only 10 cases of bilateral myelolipomas have been documented worldwide [9, 10] among about a hundred cases reported in current literature of surgically treated myelolipomas. Although there are case studies reporting their association with overproduction of adrenal hormones, adrenal myelolipomas are generally hormonally inactive and this was confirmed in our cases as well [11].

Although most adrenal myelolipomas are usually asymptomatic, abdominal/flank pain and an abdominal mass are the most common presenting symptoms. The compression of neighboring structures by large tumors (mass effect), infection, tumor necrosis and intra-tumoral hemorrhage are the main complications associated with it. Intestinal obstruction and spontaneous retroperitoneal hemorrhage are rare complications usually encountered with tumors > 10 cm in diameter [8, 12, 13].

Adrenal myelolipoma is often an “incidentaloma”, since its diagnosis is frequently based on autoptic findings or made during surgical interventions and imaging procedures performed for other purposes, as happened in our cases. However, with the advent of cross sectional imaging, the diagnosis of this neoplasm has improved remarkably, both in asymptomatic and symptomatic patients. The imaging features of myelolipoma depend on the varying proportion of fat, hematopoietic tissues, hemorrhage or calcification present within it. Calcification may be seen in these masses in around 27% of cases [6].

Although a predominantly hyperechoic adrenal mass strongly suggests the diagnosis of myelolipoma, ultrasonography is not specific and computed tomography (CT) or magnetic resonance imaging (MRI) should be obtained for further evaluation [14-16]. CT is the imaging modality of choice for these tumors because of its sensitivity, and easy availability and the appearance of myelolipoma depends on its histological composition. A well defined fatty adrenal mass with negative attenuation value (-30 to -100 HU) is almost diagnostic of myelolipoma. The presence of hematopoietic elements, intratumoral bleed, calcification and adrenal tissue accounts for non-fat density and heterogeneity. MRI is complimentary to CT in the workup of patients with adrenal masses. Furthermore, MRI also accurately depicts both microscopic and macroscopic fat using chemical shift imaging and explicit (chemically selective) fat saturation technique, respectively. In MRI, fat tissue has high signal intensity in both T1 and T2 images, whereas myeloid tissue has low signal intensity in T1 and moderate signal intensity in T2 images. Differential diagnosis includes other fat-containing adrenal masses such as teratoma, lipoma, and liposarcoma, which are less common, and even rarely angiomyolipoma, mass-forming extramedullary hematopoiesis, and adenoma.

**Figure 1:**
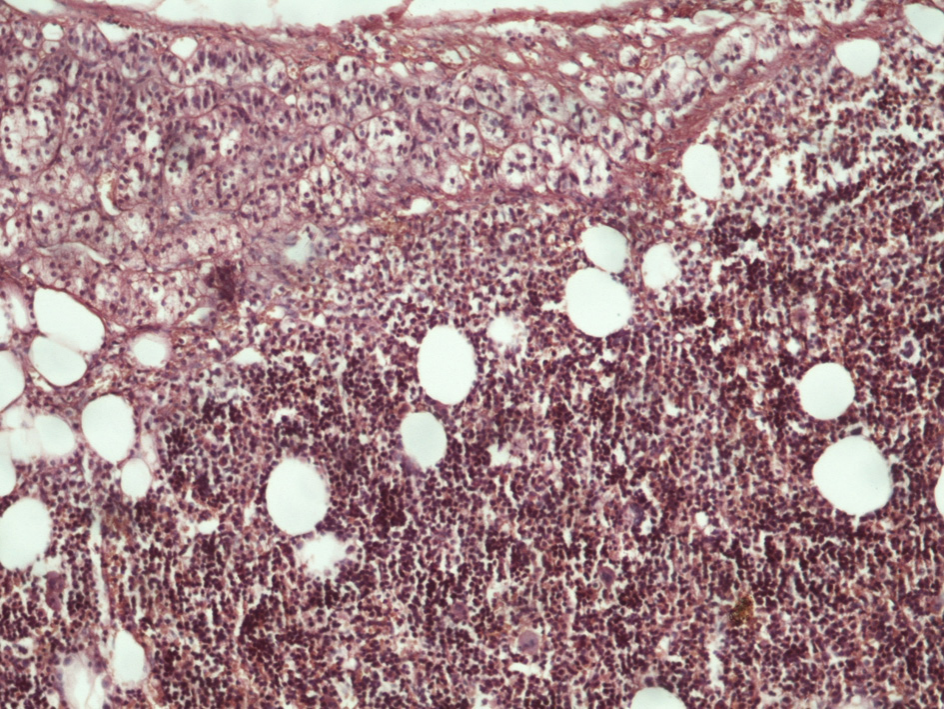
Histological section of adrenal gland showing both normal adrenal tissue and myelolipoma (hematoxylin-eosin × 120).

**Figure 2:**
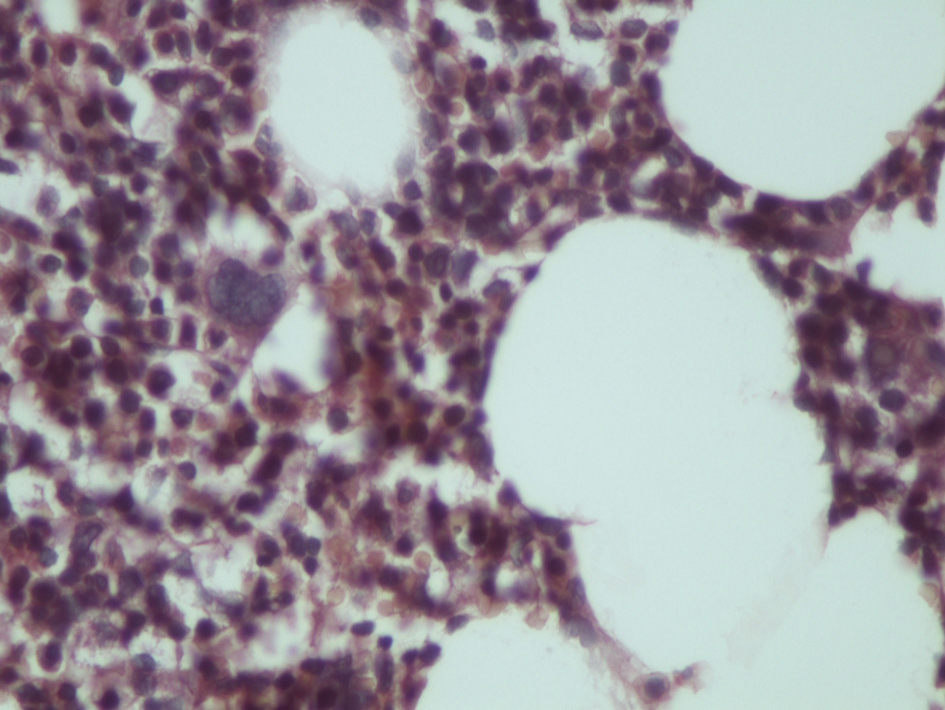
Histological section of myelolipoma indicating haemopoetic elements and lipocytes (hematoxylin-eosin × 240).

Myelolipomas are well circumscribed in gross inspection, but are rarely encapsulated. Their color varies from pale yellow to deep red or brown depending upon the relative proportion of fat and hematopoietic elements. The contour of the adrenal myelolipoma is smooth, wavy, or irregular and may shoe intermingling of cortical cells with elements of the myelolipoma. The surrounding cortical cells can be relatively normal in appearance or compressed (Fig. 1). There is a variable mixture of mature fat with hematopoietic elements, often with full representation of the major cell lines (Fig. 2). Rarely foci of ossification are seen.

Previously, most patients with myelolipoma underwent surgical resection for a suspected malignant neoplasm. Nowadays, with the frequent detection of these myelolipomas incidentally, the treatment has been a matter of much debate. Asymptomatic, small myelolipomas (< 5 cm) are treated conservatively with 6 - 12 months interval follow-up with ultrasound or CT, whereas symptomatic and large tumors (> 10 cm) should be extirpated because of risk of malignant change and spontaneous rupture with retroperitoneal bleed. With the advent of minimally invasive surgery, laparoscopic adrenalectomy has become a standard treatment for both functioning and non-functioning adrenal tumors. In the case of bilateral adrenal myelolipomas, a staged tumor removal is preferable, removing the larger one and continuing to observe the contralateral myelolipoma as long as possible in an effort to avoid adrenal insufficiency and a lifetime of steroid replacement [17].
